# Beyond single references: pangenome graphs and the future of genomic medicine

**DOI:** 10.3389/fgene.2025.1679660

**Published:** 2025-09-19

**Authors:** Denis M. Nyaga, Roan E. Zaied, Olin K. Silander, Michael A. Black, Justin M. O’Sullivan

**Affiliations:** ^1^ Liggins Institute, University of Auckland, Auckland, New Zealand; ^2^ Department of Biochemistry, University of Otago, Dunedin, New Zealand

**Keywords:** clinical diagnosis, genetic diversity, genome assembly, haplotypes, long-readsequencing, pangenome graphs, reference genomes

## Abstract

Genomic medicine relies on single reference genomes that miss crucial genetic diversity, creating diagnostic gaps that disproportionately affect underrepresented populations. Pangenome graphs, collections of diverse genomes represented as interconnected genetic paths, offer a powerful alternative to the standard reference genome approach. Pangenome-based approaches capture the spectrum of human variation, dramatically improving how we detect complex structural variants, reconstruct haplotypes, and reduce bias in genetic studies. Projects like the Human Pangenome Reference Consortium have identified hundreds of megabases of missing genetic diversity, leading to remarkable improvements in variant detection across different populations. Yet, as pangenomes grow larger and computationally complex, they become more challenging to interpret clinically, creating a trade-off between comprehensiveness and usability. This review discusses the technical and conceptual advances enabling clinical applications of pangenomes in rare disease diagnosis. Realizing the future potential of pangenome graphs in genomic medicine will require innovative implementation strategies, thorough clinical testing, and user-friendly approaches.

## 1 Introduction

Genome sequencing is transforming medicine, enabling the detection of rare genetic variants (i.e., single nucleotide variants [SNVs], structural variants [SVs], insertions and deletions [indels], copy number variants [CNVs], and short tandem repeats [STRs]) that are missed with traditional genotyping. However, standard approaches to variant discovery rely almost entirely on comparison to a single linear reference genome, which, by its nature, lacks genetic diversity and does not represent the full range of human populations (Aganezov et al., 2022; Nurk et al., 2022; Liao et al., 2023; Hickey et al., 2024; Sirén et al., 2024; Taylor et al., 2024). Over-reliance on a single reference genome is a substantial barrier to equitable, high-resolution diagnosis (Matalon et al., 2023). In this review, we argue that pangenomes (i.e., collections of genomes) are not merely an incremental improvement but, together with graph-based genome encoding (i.e., the storage of genomic data as haplotype paths), constitute a disruptive paradigm shift that will render current variant discovery pipelines in genomic medicine obsolete. Ironically, as more pangenomes are built with increasingly large collections of genetic variations, it will become harder for clinicians and researchers to understand and use them effectively. As such, new approaches are required to enable rapid, interpretable pangenome queries.

## 2 The linear reference paradox

The Genome Reference Consortium (GRC) currently maintains two primary human reference assemblies: GRCh37 (hg19, released 2009) (Church et al., 2011) and its successor GRCh38 (hg38, published 2013) (Schneider et al., 2017). The GRCh38 assembly is a composite of unphased single haplotypes, with about 70% derived from a single individual, 23% from ten, and 7% from over fifty additional sources (Ballouz et al., 2019). These two reference genomes serve as critical foundations for genomic research, enabling clinical, comparative, developmental, population, and disease analyses (Lowy-Gallego et al., 2019; Abascal et al., 2020; Collins et al., 2020; GTEx Consortium, 2020; Taliun et al., 2021; Aganezov et al., 2022; Nassar et al., 2023; Reis et al., 2023; Mahmoud et al., 2024; Nyaga et al., 2024). The power that the incorporation of these references into biological studies brings has been unequivocally demonstrated, including through the identification of the genetic and molecular basis of rare diseases (Lunke et al., 2023; Nyaga et al., 2024; Sinha et al., 2025).

**FIGURE 1:**
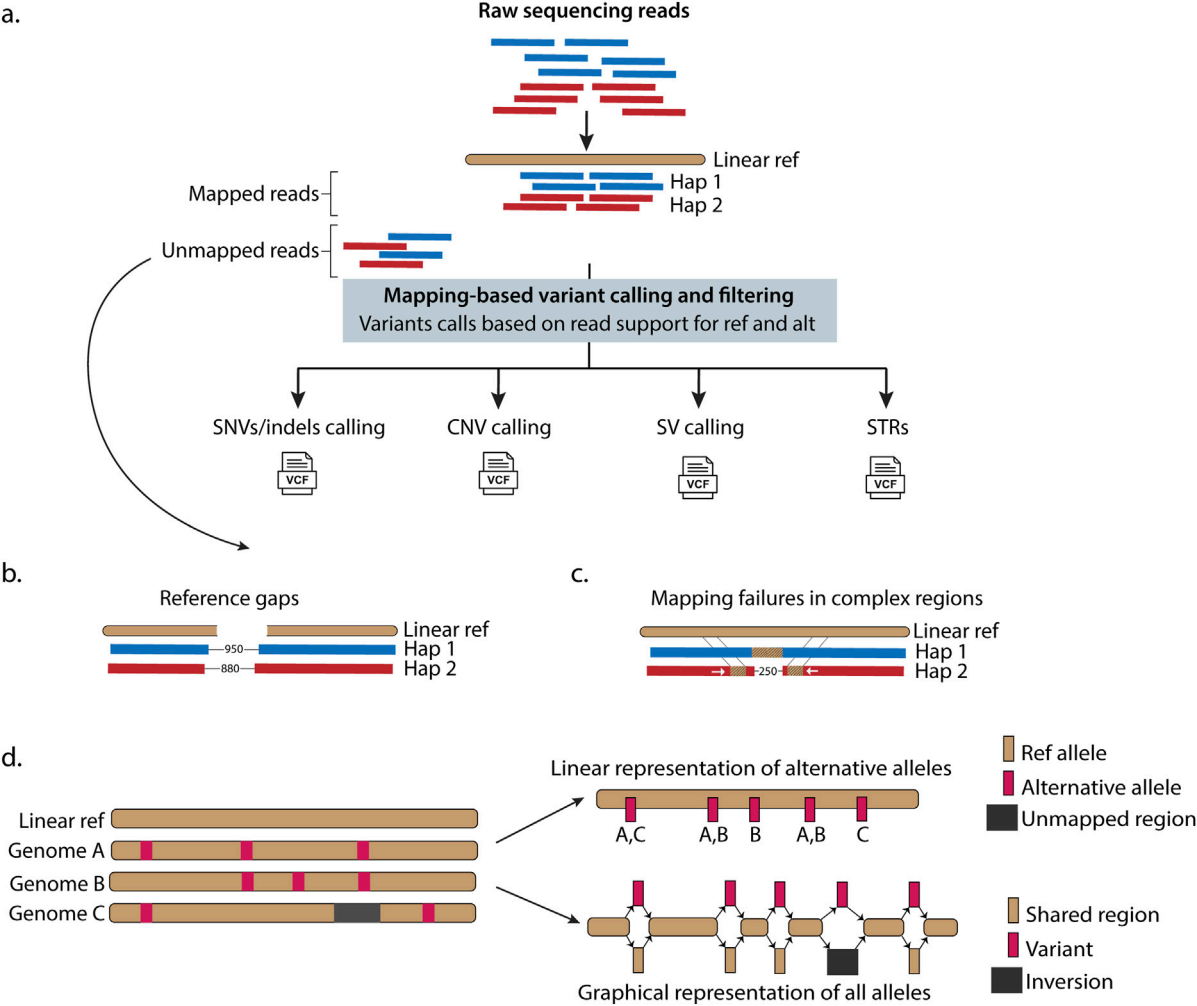
Linear reference-based genomic analysis has inherent limitations in alignment, variant detection, and representing population genetic diversity. **(a)** Raw sequencing reads from paired haplotypes are aligned to a linear reference genome, introducing inherent bias when genomic regions diverge significantly from the reference. Successfully aligned reads undergo variant detection for SNVs, small indels, SVs, CNVs, and STRs based on the read depth support for the linear reference (ref) and alternative (alt) sequence. However, variants in highly divergent regions remain undetected when reference genome sequencing reads fail to map to the linear reference due to reference genome gaps **(b)** or complex variations within genomic regions (such as an insertion on hap (haplotype) 1 and two inverted translocations with a deletion on hap (haplotype 2) **(c)**. **(d)** Linear references inadequately represent population genetic diversity. Linear models represent variations relative to the reference while graph-based models enable direct all-to-all genome comparisons, capturing complete sequence relationships. Graphs models efficiently represent SVs (such as the inversion shown in dark grey) that linear models miss. Hap - haplotypes, ref - reference, VCF - variant call format file.

GRCh37 and GRCh38 are not and have never been fixed entities. Rather, the GRC has continually worked to improve these assemblies by implementing patches, fixes, and alternate scaffolds to represent allele diversity. For example, the transition from GRCh37 to GRCh38 included approximately 100 Megabases (Mb) of improvements, particularly in immune-related regions (Schneider et al., 2017). Despite these efforts, the lack of ancestral diversity within these references remains a considerable limitation (Figure 1), particularly in clinical settings. For example, it is possible that diagnostic *de novo* pathogenic variants remain undetected because they lie outside the reference structure in the gaps (i.e., 7% or 210 Mb of its primary chromosome, unknown sequences [151 Mb], or computationally simulated regions [59 Mb] that are present in the reference structure (Figures 1A–C) (Aganezov et al., 2022; Nurk et al., 2022). Additionally, GRCh38 includes alternative contigs (i.e., continuous stretches of DNA sequence representing alternative haplotype diversity), which can lead to variant calling errors (Jia et al., 2020; Li H. et al., 2021; Aganezov et al., 2022) and biased variant interpretation, particularly towards insertions and deletions (indels) (Church et al., 2011; Schneider et al., 2017; Pan et al., 2019; Li H. et al., 2021). For example, mishandling alternative scaffold inclusion resulted in incorrect reports of genetic variation in 641 genes in UK Biobank exome data (Jia et al., 2020).

Despite its limitations, it must be acknowledged that substantial progress has been made in the continual development of the reference human genome. Since the first draft of the human genome was published in 2001, the quality (i.e., accuracy of base calling and assembly) and contiguity (i.e., the length of continuous DNA sequences without gaps) have improved substantially. Indeed, the initial human genome was incomplete and highly fragmented, consisting of more than 150,000 contigs, with just over half the genome represented in contigs greater than 50 Kilobases (Kb) (Church et al., 2011). The recent incorporation of long reads, which can span multiple Kb in a single continuous read, has allowed researchers to create a telomere-to-telomere genome assembly (T2T) (Rautiainen et al., 2023). The T2T-CHM13v2.0 human reference genome (Cai et al., 2020; Nurk et al., 2022) is a near-gapless, ‘error-free’ telomere-to-telomere assembly consisting of over 3.0 Gigabases (Gb) of fully resolved sequence that is contiguous across all autosomes and chromosome X (except for the highly repetitive ∼9 Mb sequence from ribosomal DNA arrays) for a haploid human genome (Nurk et al., 2022).

The complete T2T genome assembly has contributed to the successfully resolved previously difficult-to-sequence regions, including the short arms of all five acrocentric chromosomes, centromeric repeats and segmental duplication (Nurk et al., 2022). This has enabled researchers to study genetic variation across these complex regions (Jeong et al., 2025). Despite representing only a single human haplotype, the T2T-CHM13v2.0 reference has already contributed to improvements in genomic variant discovery. For example, the assembly has enabled the discovery of over 2 million additional SNVs in regions missing from GRCh38 and has improved CNV detection across the 1000 Genomes Project samples (Aganezov et al., 2022; Nurk et al., 2022). In addition, researchers have attempted to genotype population-level SVs by utilizing long-read sequencing in diverse genomes and mapping these reads to the T2T assembly, identifying a large number of novel SVs (Reis et al., 2023; Gustafson et al., 2024; Logsdon et al., 2025; Schloissnig et al., 2025). However, even with these significant advances, the T2T-CHM13v2.0 assembly does not fully represent the genetic diversity of the human population, as variation can only be comprehensively studied in the context of multiple populations, not just by comparison to a single reference (Figure 1D) (Garrison et al., 2018; Eizenga et al., 2020; Sirén et al., 2021; Wang et al., 2022; Liao et al., 2023).

The standardized coordinate systems provided by reference genomes are essential for communication and coordinated analysis across the scientific and medical communities (Ballouz et al., 2019). However, no single reference genome can fully represent human diversity. This and the other inherent limitations of the GRCh38 and T2T linear reference assemblies are becoming particularly obvious as individualized approaches to medicine and rare disease increase. This is particularly true for patients of non-European ancestry, who experience substantially lower diagnostic rates. One indication of this disparity is an observed ∼23% increase in the burden of variants of uncertain significance (VUS) compared to individuals of European ancestry (Dawood et al., 2024). Such missed diagnoses translate into increased morbidity, highlighting the importance of the inequalities that arise from the clinical use of a single human genome reference sequence (Green et al., 2023; Matalon et al., 2023). To address this, clinical studies must transition to use collections of genome assemblies as part of ancestrally diverse pangenome-based approaches that are backwardly compatible with published knowledge.

## 3 Graph-based pangenomes as next-generation references

**FIGURE 2:**
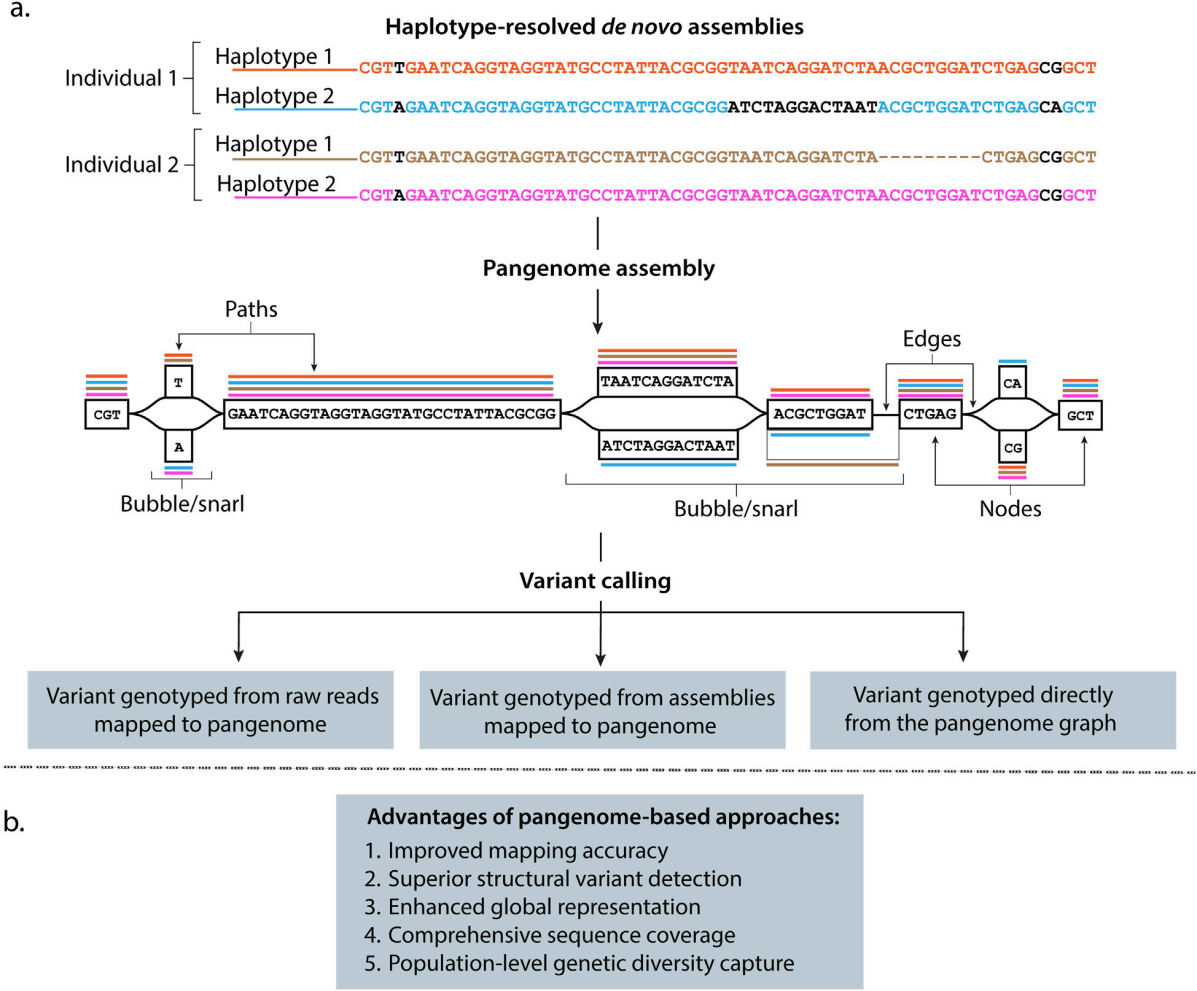
Graph-based approaches overcome limitations of linear genome representations while ensuring backward compatibility and preserving analytical continuity. **(a)** High-quality haplotype-resolved *de novo* assemblies provide the foundation for building pangenome frameworks. Pangenome graphs represent assembled genomes from individuals (i.e., Individual 1, Individual 2, … , Individual n) as embedded haplotype paths (colored lines) where nodes contain DNA sequences linked by directional connections, with edges illustrating interconnections between DNA sequences. Individual haplotypes follow specific paths (colored lines) through these nodes, while branching regions called “bubbles” or “snarls” capture genetic variations (nucleotides colored in black), including SVs, where genomes differ. For example, the inversion in haplotype 2 from Individual 1 and the deletion in haplotype 1 from Individual 2 are represented in the graph as bubbles or snarls with different paths. This structure preserves genetic diversity from all contributing genomes. Variant detection from pangenomes can be performed by mapping sequencing reads directly to the graph, aligning new assemblies to the pangenome reference, or analyzing variants already represented within the graph structure. **(b)** Advantages of using pangenome-based approaches instead of linear references in genomic analysis.

The concept of ‘pangenome’ was initially introduced in 2000 by Sigaux, who applied it to describe a comprehensive database containing genomic and transcriptomic changes found in tumors, healthy cells and experimental systems (Matthews et al., 2024). However, the term has evolved to the current graph reference (as reviewed by (Marschall et al., 2018) to describe a set of whole-genome assemblies from multiple individuals that are used together as a reference or analyzed collectively (Marschall et al., 2018; Eizenga et al., 2020; Wang et al., 2022; Liao et al., 2023). These collections of multiple genomes enable the inclusion of variation from human populations, especially when they include samples from individuals underrepresented in previous genetic studies. As such, pangenomes offer a promising alternative to single linear reference assemblies for studying genetic variation (Figure 2A) (Wang et al., 2022; Gao et al., 2023; Liao et al., 2023). However, pangenomes also risk creating new inequities if not carefully implemented. If pangenomes are built predominantly from well-resourced populations or lack diverse ancestral representation, they will perpetuate existing biases despite appearing inclusive. The technical and cost implications of pangenome initiatives should not be overlooked. For example, the substantial amount of genetic data required to build ancestrally diverse pangenomes could restrict access for researchers and clinicians in resource-constrained settings (Gong et al., 2023; Liao et al., 2023). Additionally, as these pangenomes grow larger, they become computationally demanding, requiring extensive memory to process complete graphs, complex sorting algorithms, specialized visualization tools (e.g., Optimized Dynamic Genome/Graph Implementation [ODGI] (Guarracino et al., 2022)), and sophisticated indexing methods, which consequently make clinical interpretation challenging (Gong et al., 2023). This creates a trade-off between comprehensiveness and usability, potentially widening the genomic equity gap between well-resourced and under-resourced communities.

Pangenome construction relies on generating high-quality, haplotype-resolved genome assemblies. Long-read sequencing technologies have enabled the generation of these haplotype-resolved genomes, where maternal and paternal chromosome segments are distinctly identified. These precisely assembled and haplotype-resolved genomes can be organized into graph-based data structures, using two dominant computational approaches: 1) sequence graphs (Garrison et al., 2018; Hickey et al., 2020; Hickey et al., 2023; Sirén et al., 2021; Groza et al., 2024) (e.g., minigraph (Li et al., 2020); and 2) de Bruijn graphs (Iqbal et al., 2012; Minkin et al., 2017; Holley and Melsted, 2020) (Box 1), that efficiently compress and index the sequence information while maintaining an intuitive coordinate system for genetic variant identification (Garrison et al., 2018; Hickey et al., 2020; Sirén et al., 2021; Liao et al., 2023).

However, representing complex structural variants in pangenomes remains challenging. While efforts exist to unify coordinates using the reference graphical fragment assembly (rGFA) format, which provides a stable coordinate system indicating the origin of segments from linear genomes, no straightforward method exists for representing complex SVs in VCF files (Li et al., 2020). This is particularly problematic for nested variants (i.e., bubbles within bubbles) or variants occurring only on alternative haplotypes (Li et al., 2020). This complex problem is compounded by the requirement for a linear reference backbone, which reintroduces the very biases that pangenomes are designed to eliminate. Despite these challenges, the incorporation of haplotype-resolved genome assemblies into pangenomes has improved upon linear references, while the stable coordinate system ensures backward compatibility, thereby preserving analytical continuity (Garrison et al., 2018; Sirén et al., 2021; Wang et al., 2022; Liao et al., 2023; Secomandi et al., 2025).

Box 1 **Sequence and de Bruijn graphs: a quick summary**

Sequence graphs have clear advantages over de Bruijn graphs for clinical application due to their stable coordinate systems and support for complex SVs. This enables precise backwardly compatible connections between graph structures and biological features for accurate variant identification (Li et al., 2020; Andreace et al., 2023; Chin et al., 2023; Hickey et al., 2023; Groza et al., 2024).

Sequence graphs:

• Nodes represent variable length DNA sequences (or reverse complement, depending on traversal direction). Edges represent interconnections between DNA sequences (Figure 2a).
• “Bubbles” or “snarls” are defined as divergent paths in the graph where sequences from different individuals branch apart and then reconverge, thereby connecting common head and tail nodes and representing genetic variations (Figure 2a) (Onodera et al., 2013; Paten et al., 2018; Eizenga et al., 2020; Dabbaghie et al., 2022; Secomandi et al., 2025).
• Variation graphs consist of all possible sequences from a population and embed sample haplotype sequences as navigable paths.
• Key advantage: provides a stable coordinate system that remains consistent regardless of methodology of the graph construction (Li et al., 2020; Hickey et al., 2023; Hickey et al., 2024; Sirén et al., 2024).
• Limitation: scalability is a significant limitation for sequence/variation graphs.
• Enable precise alignment, annotation, and comparative analysis across variation graphs and linear reference genomes (Figure 2b).

De Bruijn graphs:

• Nodes represent fixed-length sequences (*k*-mers) while edges represent interconnections between DNA sequences (Iqbal et al., 2012; Andreace et al., 2023).
• Colored de Bruijn graphs enhance pangenome analysis by assigning sample- or population-specific identifiers, enabling efficient population-scale genomic studies (Iqbal et al., 2012; Holley and Melsted, 2020).
• Limitations: these graphs struggle to resolve repetitive genomic regions due to their reliance on fixed *k*-mer lengths (Bankevich et al., 2022), and cannot be effectively built from noisy sequencing reads (Nie et al., 2024)
• Computational efficiency is improved through using compact data structures and the Burrows-Wheeler transform (BWT) (Baier et al., 2016).
• Challenges persist in maintaining connections between graph structures and the original sequence coordinates, which is essential for reference pangenome applications (Li et al., 2020; Hickey et al., 2023).

Early attempts to build a human pangenome identified novel sequences absent from the standard reference genome assembly. For example, the African Pangenome Project uncovered >290 Mb of novel contigs from 910 individuals of African descent (Sherman et al., 2019) Similarly, the HUPAN initiative, which constructed the first Chinese pangenome from 275 individuals, revealed 29.5 Mb of population-specific novel sequences (Duan et al., 2019) Li et al. also utilized deep sequencing data from 486 Han Chinese individuals to build a pangenome that contained 276 Mb of sequences absent from the current human reference (Li Q. et al., 2021).

The Human Pangenome Reference Consortium (HPRC) used long-read, phased, diploid assemblies to create a more inclusive reference (Wang et al., 2022; Liao et al., 2023). The first HPRC release contained 47 phased diploid genomes representing 94 haplotypes. This has subsequently expanded to include phased haplotypes from 232 individuals in the most recent release. The genomes that were included in the HPRC pangenome were initially selected from the 1000 Genomes Project (1KGP) to represent diverse ancestries: 24% African, 30% Americas, 18% East Asian, 28% South Asian (https://humanpangenome.org/samples/) (Wang et al., 2022). To improve global representation, the HPRC is expanding beyond these initial samples to incorporate 700 haploid genomes from cohorts that include the BioMe Biobank and African American individuals to maximize diversity and create a truly representative global pangenome reference (Wang et al., 2022). This population-level approach has improved SV detection, identifying an average of over 29,000 SVs per individual compared to fewer than 16,000 SVs when using linear reference (Groza et al., 2024; Schloissnig et al., 2025). Additionally, this approach has enhanced genotyping accuracy and reduced the reference bias inherent in traditional genomic analysis methods (Hickey et al., 2020; Sirén et al., 2021; Sirén et al., 2024; Lee et al., 2022; Liao et al., 2023).

## 4 Building, manipulating and querying pangenome graphs

While pangenome graphs offer powerful representations of genomic diversity, their practical application depends on our ability to effectively construct, query, and analyze these complex data structures. This involves building graphs from multiple assemblies, extracting specific genomic regions, performing comparative analyses, and integrating functional annotations. However, efficient pangenome graph manipulation requires substantial computational resources and technical expertise (Garrison et al., 2024; Secomandi et al., 2025). To address these challenges, methods have been developed for *de novo* genome assembly, collecting and mapping assemblies into pangenomes, and annotating pangenome graphs (Table 1).

**TABLE 1:** Popular open-source tools for *de novo* genome assembly, construction and annotation of pangenomes.

Tool name (Year) *GitHub URL*	Description
Genome assembly	
Canu (2017) https://github.com/marbl/canu	Assembly tool for noisy long reads (i.e., PacBio CLR and ONT reads) into a graphical fragment assembly that can be integrated with complementary phasing and scaffolding methods
Flye (2019) https://github.com/fenderglass/Flye	A de novo assembler for long reads (i.e., PacBio CLR, HiFi and ONT reads) into genomes using repeat graph
GoldRush (2023) https://github.com/bcgsc/goldrush	GoldRush produces a “golden path” of long reads (i.e., PacBio CLR and ONT reads) with ∼1 fold coverage, which are then polished and scaffolded into the final assembly
Hifiasm (2021) https://github.com/chhylp123/hifiasm	Constructs haplotype-resolved assemblies from accurate PacBio HiFi reads and ultralong ONT reads
La Jolla Assembler https://github.com/AntonBankevich/LJA	A tool for genome assembly from PacBio HiFi reads based on de Bruijn graphs
MECAT2 (2019) https://github.com/xiaochuanle/MECAT2	An ultra-fast and accurate mapping, error correction and de novo assembly tool for long reads (i.e., PacBio CLR, HiFi reads)
Miniasm (2016) https://github.com/lh3/miniasm	A fast OLC-based de novo assembler for noisy long reads (i.e., ONT reads) into an assembly graph in the GFA format
NECAT (2021) https://github.com/xiaochuanle/NECAT	An error correction and de novo assembly tool for noisy long reads (i.e., ONT reads)
NextDenovo (2024) https://github.com/Nextomics/NextDenovo	A string graph-based de novo assembler for long reads (i.e., PacBio CLR and ONT reads) that uses a “correct-then-assemble”
PECAT (2024) https://github.com/lemene/PECAT	A haplotype-aware correction and assembly tool for long noisy reads (i.e., PacBio CLR and ONT reads)
Peregrine-2021 (2022) https://github.com/cschin/peregrine-2021	A genome assembler designed for long reads that have good enough accuracy (i.e., PacBio CLR and ONT reads)
Raven (2021) https://github.com/lbcb-sci/raven	A de novo genome assembler for long uncorrected reads (i.e., PacBio CLR and ONT reads)
Rust-mdbg (2021) https://github.com/ekimb/rust-mdbg/	An ultra-fast minimizer-space de Bruijn graph implementation, geared towards the assembly of long and accurate PacBio HiFi reads
Shasta (2020) https://github.com/paoloshasta/shasta	A de novo assembler for long reads optimized for ONT reads
SMARTdenovo (2021) https://github.com/ruanjue/smartdenovo	A de novo assembler for long reads (i.e., PacBio CLR and ONT reads) into an assembly from all-vs-all raw read alignments without an error correction
Verkko (2023) https://github.com/marbl/verkko	A hybrid telomere-to-telomere genome assembly pipeline of accurate long reads (PacBio HiFi, ONT Duplex, and HERRO corrected ONT simplex reads) and ONT ultra-long reads
Wtdbg2 (2020) https://github.com/ruanjue/wtdbg2	A de novo sequence assembler for noisy long reads (i.e., PacBio CLR and ONT reads) into FBG
Pangenome construction	
Bifrost (2020) https://github.com/pmelsted/bifrost	Tool for parallel construction, indexing and querying of colored and compacted de Bruijn graphs
MEMO (2025) https://github.com/StephenHwang/MEMO	A pangenome indexing method based on maximal exact matches between genomes
Minigraph (2020) https://github.com/lh3/minigraph	Tool for sequence-to-graph mapping, with incrementally sequence mapping to existing graphs, and variant calling.
Minigraph-Cactus (2023) https://github.com/ComparativeGenomicsToolkit/cactus	A pangenome graph construction toolkit
Pangene (2024) https://github.com/lh3/pangene	Constructs pangenome gene graphs, with nodes representing marker genes and edges between two genes indicating their genomic adjacency on input genomes
Pangenome (2025) https://github.com/nf-core/pangenome	A bioinformatics best-practice analysis pipeline that renders a collection of sequences into a pangenome graph
Pannagram (2025) https://github.com/iganna/pannagram	A tool for constructing pan-genome alignments, analyzing SVs, and translating annotations between genomes
PanPipes (2022) https://github.com/USDA-ARS-GBRU/PanPipes	An end-to-end pipeline for pan-genomic graph construction and genetic analysis
PanTools (2025) https://git.wur.nl/bioinformatics/pantools	A toolkit for building pangenomes from genomes using de Bruijn graphs and constructing pan-proteomes from proteins
Pggb (2025) https://github.com/pangenome/pggb	Builds pangenome variation graphs from a set of input sequences
Psvcp (2023) https://github.com/wjian8/psvcp_v1.01	A tool for pangenome construction and population structure variation genotype calling pipeline
Pangenome annotation	
GrAnnot (2025) https://forge.ird.fr/diade/dynadiv/grannot	An annotation transfer tool for pangenome graphs that transfers linear genome annotations to a pangenome graph
PanTools (2025) https://git.wur.nl/bioinformatics/pantools	Constructs and expands the annotation layer of an existing pangenome using genomic features like genes, mRNAs, proteins, tRNAs from GFF files

ONT, Oxford Nanopore Technologies; PacBio - Pacific Biosciences; CLR, continuous long reads; HiFi - high-fidelity; HERRO, Haplotype-aware ERRor cOrrection; FBG, fuzzy Bruijn graph; GFA, graphical fragment assembly text format describing a set of sequences and their overlap; OLC, Overlap-Layout-Consensus paradigm; GFF, General Feature Format; tRNA, transfer RNA; mRNA, messenger RNA; Year - represents year of publication or the year of the latest version available on GitHub.

Pangenome graph builders such as PanGenome Graph Builder (pggb), Minigraph-Cactus, and TwoPaCo can deal with mammalian-sized (∼3 Gb) assemblies. Minigraph constructs specialized pangenome graphs through iterative sequence alignment to reference templates. Human pangenome projects have utilized these tools at varying scales: Pantools (7 genomes (Sheikhizadeh et al., 2016); Minigraph-Cactus and pggb (94 single chromosomes (Hickey et al., 2023; Liao et al., 2023); TwoPaCo (100 simulated genomes (Minkin et al., 2017); and Minigraph (94 (Li et al., 2020) and 574 (Groza et al., 2024) haplotype-resolved assemblies). Recently, the HPRC released a draft human reference pangenome constructed using pggb and Minigraph-Cactus pipelines (Liao et al., 2023). As more diverse genomes are assembled *de novo*, the resulting pangenome references will progressively capture the full spectrum of human genomic diversity, ultimately enhancing our ability to detect and interpret rare and clinically relevant variants in precision medicine applications.

### 4.1 Advantages of genome graphs for variant calling

Traditional variant calling relies on aligning reads to a single reference genome (i.e., GRCh37, GRCh38, or more recently, T2T-CHM13). However, linear reference approaches struggle with regions where individuals differ substantially from the reference, including CNVs, SVs, large indels, and highly polymorphic regions (e.g., killer immunoglobulin-like receptor [KIR] and human leucocyte antigen [HLA] loci) (Kulski et al., 2022; Olson et al., 2023). To overcome these challenges, researchers have developed methods to map reads to pangenome references using graph-based structures that incorporate variants from diverse individuals as alternative paths, with alignments often converted back to linear references for compatibility with conventional variant-calling tools (Table 2).

**TABLE 2:** A list of popular open-source tools for pangenome-based variant genotyping.

Tool name (Year) *GitHub URL*	Description
Ctyper (2024) https://github.com/ChaissonLab/Ctyper	A pangenome allele-specific and copy number specific genotyping tool
DeepVariant (2025) https://github.com/google/deepvariant	A deep learning-based variant caller for alignments (BAM or CRAM) and pangenome graphs
Graphtyper2 (2019) https://github.com/DecodeGenetics/graphtyper	A graph-based variant caller capable of genotyping population-scale short read data sets
Minigraph (2020) https://github.com/lh3/minigraph	Tool for sequence-to-graph mapping, with incrementally sequence mapping to existing graphs, and variant calling
Minigraph-Cactus (2023) https://github.com/ComparativeGenomicsToolkit/cactus	A graph construction and variant genotyping toolkit
PanGenie (2024) https://github.com/eblerjana/pangenie	A short-read genotyper for SNPs, indels and SVs represented in a pangenome graph
Paragraph (2019) https://github.com/Illumina/paragraph	A graph-based structural variant genotyping tool for short-read sequence data
PHI (2024) https://github.com/at-cg/PHI	A pangenome-based genotyping method from low-coverage sequencing data (short-reads or long-reads)
SVarp (2024) https://github.com/asylvz/SVarp	A tool to discover haplotype resolved SVs on top of a pangenome graph reference using long sequencing reads
Varigraph (2025) https://github.com/JiaoLab2021/varigraph	A pangenome graph-based variant genotyper for diploid and polyploid genomes
Vg (2020) https://github.com/vgteam/vg	A pangenome-based SV genotyping tool

BAM, Binary Alignment Map of genome sequencing data; CRAM, Compressed Reference-oriented Alignment Map of genome sequencing data; Year - represents year of publication or the year of the latest version available on GitHub.

Recent advances by the HPRC have significantly improved variant detection through graph-based references constructed from long-read high-fidelity (HiFi) reads that provide per-base accuracy of 99.9%. Specialized tools further enhance variant calling (e.g., Giraffe-DeepVariant) and variant genotyping (e.g., PanGenie), particularly for large indels, SVs and variations in highly polymorphic regions previously problematic in GRCh38 (Table 2) (Li et al., 2020; Sirén et al., 2021; Ebler et al., 2022). The Minigraph-Cactus pangenome pipeline represents a significant computational advancement by combining fast assembly-to-graph mapping with an improved base aligner and including all SNVs and small indels in the pangenome (Hickey et al., 2023). This approach constructs nucleotide-resolution pangenome graphs through a two-stage process: first extracting SVs from each of hundreds of haplotype-resolved assemblies, then using these variants as alignment anchors to generate a comprehensive base-level graph. The resulting graph represents variation at all resolutions (i.e., from SNVs to complex SVs such as inversions) (Hickey et al., 2023).

Minigraph-generated pangenome graphs have improved short-read and long-read mapping, variant calling, and SV genotyping (Hickey et al., 2023). For example, minigraph has recently been employed to identify 200,000 unique SVs from a pangenome graph of 574 assemblies, outperforming standard methods (Groza et al., 2024). However, despite multiple tools promising perfect recall for complex SVs, few clinical labs have validated these graph-based callers on patient cohorts. Therefore, randomized trials testing graph genotypers are required to determine if they improve the detection of clinically relevant indels and variants.

### 4.2 Clinical impact and research applications of pangenome graphs

Pangenomes represent a paradigm change in the conceptualization and analysis of human genetic diversity (Eggertsson et al., 2019; Hickey et al., 2020; Sirén et al., 2021; Ebler et al., 2022; Lee et al., 2022; Tetikol et al., 2022; Liao et al., 2023; Groza et al., 2024; Secomandi et al., 2025). The potential impact of the use of pangenomes is particularly notable in applications that incorporate accurate haplotype reconstruction into the diagnosis of rare disorders (Abondio et al., 2024; Groza et al., 2024) and complex SV interpretation (Hickey et al., 2020; Ebler et al., 2022; Lee et al., 2022; Tetikol et al., 2022; Groza et al., 2024). However, simply aggregating more genomes will not solve the fundamental problem of missing diversity, as bigger is not always better. Clinically, strategic sampling and generation of pangenomes from related individuals (e.g., trios–mother, father, child, or siblings) may yield more clinically actionable variants per unit (e.g., terabase) of sequencing, particularly in genomic regions that are poorly captured by standard linear reference genomes. In addition, by moving away from short-read sequencing, which suffers from a limited ability to resolve complex SVs and repetitive regions, pangenome efforts will improve the clinical utility of these genomic features (Abondio et al., 2024; Groza et al., 2024).

#### 4.2.1 Accurate haplotype reconstruction

Many genome assembly methods collapse heterozygous alleles that are present in diploid organisms, erasing heterozygous variation and potential misrepresentation in regions of significant haplotype divergence (Dilthey et al., 2015; Li et al., 2020; Ebler et al., 2022; Chin et al., 2023; Matthews et al., 2024; Secomandi et al., 2025). This limitation is particularly problematic in genomic regions with high sequence diversity or complex SVs, such as the major histocompatibility complex (MHC) region on chromosome 6 that encodes the classical human leucocyte antigen alleles (Dilthey et al., 2015; Li et al., 2020).

However, a diversely sampled pangenome graph for the highly complex MHC region allows inference of haplotypes using only short-read sequencing data even in regions that were previously difficult to characterize accurately (Dilthey et al., 2015). Notably, approximately 1% of the human genome is poorly represented by linear references, including gene-dense loci containing the olfactory receptors and ubiquitin-specific peptidases (Dilthey et al., 2015). As such, incorporating alternative sequence variants through graph-based models significantly enhances our ability to reconstruct accurate genomic representations. Thus, the development of population reference graphs across the MHC locus highlights the broader potential impact of graph-based methods in regions of high sequence diversity.

#### 4.2.2 Precise detection and phasing of genetic variants

The success of pangenome-based variant calling, however, depends critically on both variant characteristics and sequencing technology, with effectiveness varying significantly by variant size when using short-read sequencing data (Eizenga et al., 2020). For SNVs and small indels, pangenomes offer modest improvements in detection accuracy (Eizenga et al., 2020; Li et al., 2020; Hickey et al., 2023; Hickey et al., 2024; Secomandi et al., 2025). However, pangenome graphs demonstrate substantial improvement in genotyping SVs (e.g., over 10% increase in number of SVs detected), addressing a key limitation of traditional approaches (Hickey et al., 2020; 2024; Li et al., 2020; Sirén et al., 2021). This improvement stems from a fundamental technical constraint for short-read sequencing. Specifically, short reads can encompass small variants entirely but fail to span larger structural changes (Hickey et al., 2020; Hickey et al., 2024; Sirén et al., 2021). By contrast, pangenome graphs incorporate SVs into their framework, significantly improving variant detection, even from short-read data, compared to the currently used single-reference methods (Ebler et al., 2022; Groza et al., 2024). For example, graphs built from haplotype-resolved assemblies can harness short-read k-mer patterns to identify previously undetectable SVs (Groza et al., 2024). Additionally, some pangenome graph representations (e.g., de Bruijn) are capable of SV detection without requiring a reference genome of any type, offering a flexible alternative for variant discovery (Iqbal et al., 2012).

Pangenomes have proven valuable for population-scale variant detection (Tetikol et al., 2022; Hickey et al., 2024). For example, the pan-African (Tetikol et al., 2022) and the Chinese pangenomes (Gao et al., 2023) have substantially improved variant detection accuracy compared to traditional linear reference approaches. The effectiveness of these graphs is influenced by two key factors: nucleotide diversity within populations and the level of absolute divergence from linear reference sequences (Tetikol et al., 2022; Hickey et al., 2024). This is particularly relevant for highly diverse populations like Africans (i.e., >290 Mb of novel contigs), or groups with significant archaic admixture, such as some individuals from Australo-Melanesian populations, who may retain Denisovan haplotypes over 250 kb in length (Jacobs et al., 2019; Sherman et al., 2019; Tetikol et al., 2022).

Population-specific graphs that incorporate cohort-specific information enable the identification of functionally important variants within coding regions that are missed by standard variant calling pipelines (Ebler et al., 2022; Tetikol et al., 2022; Gao et al., 2023; Groza et al., 2024; Hickey et al., 2024). Notably, these graphs provide improvements in sensitivity and specificity typically achieved by calling variants jointly from cohorts, but without requiring simultaneous processing of all cohort samples. Thus, they represent a computationally efficient solution for large-scale genomic studies (Eggertsson et al., 2019; Ebler et al., 2022; Tetikol et al., 2022; Gao et al., 2023; Groza et al., 2024; Hickey et al., 2024; Wu et al., 2024).

#### 4.2.3 Exemplar application of pangenome graphs to rare disease diagnosis

Recent application of pangenome graph approaches has demonstrated their promise in rare disease diagnosis (Chin et al., 2023; Gao et al., 2023; Groza et al., 2024). Groza et al. established a practical framework for clinical implementation (Groza et al., 2024). Their graph-based analysis of 574 rare disease cases identified >200,000 unique and >500,000 shared SVs and ∼1,000 rare (MAF <0.01) coding variants (Groza et al., 2024). The pangenome approach proved particularly useful in complex genomic regions where traditional methods fail, enabling the identification of a previously undetectable diagnostic variant in *KMT2E* associated with macrocephaly, hypotonia, and developmental delay. These results highlight the potential of pangenome graphs to enhance diagnostic yields through improved variant detection and prioritization of candidate SVs, while providing a scalable resource for the rare disease community (Groza et al., 2024).

## 5 Discussion and future perspectives

The transition from linear reference genomes to pangenome graphs represents a transformative paradigm shift in how we conceptualize and analyze human genetic diversity, addressing fundamental limitations in variant detection and population representation. Through initiatives like the HPRC and population-specific pangenome projects, we can access sophisticated graph-based frameworks that capture hundreds of megabases of previously missing genetic diversity. Tools such as Minigraph-Cactus (Hickey et al., 2023) and specialized variant callers have demonstrated remarkable improvements in structural variant detection, accurate haplotype reconstruction, and genotyping accuracy across diverse populations, improving diagnostic accuracy for rare genetic disorders and reducing reference bias in underrepresented populations. However, significant challenges remain in computational scalability, clinical validation, interpretability, and user accessibility.

Realizing the transformative potential of pangenomes requires several key advances, which are currently being developed and researched around the world. Specifically: 1) strategic selection of samples to ensure genetic diversity by prioritizing quality over quantity in genome selection, as demonstrated by the HPRC initiative (Wang et al., 2022); 2) leveraging long-read sequencing technologies and tools that facilitate T2T genome assembly, as demonstrated by (Rautiainen et al., 2023), enabling haplotypes to be added to the pangenome graphs; 3) developing efficient algorithms for building, genotyping and annotating genetic variants from pangenome graphs, with tools such as Minigraph-Cactus (Hickey et al., 2023) being continuously improved for computational efficiency and GrAnnot (Marthe et al., 2025) ensuring annotation of sequences within these graphs; 4) conducting rigorous validation for variant detection in clinical cohorts, such as benchmarking variant calling using standards set by the Global Alliance for Genomics and Health (GA4GH (Krusche et al., 2019)) and including Genome in a Bottle (GIAB (Zook et al., 2014)) samples in pangenomes; and 5) creation of simplified analytical workflows that create equitable detection of clinically relevant variants for routine genomic medicine. Additionally, the development of splice-aware population-level RNA sequencing analysis algorithms has enabled precise quantification of haplotype-specific transcript expression (Sibbesen et al., 2023; Secomandi et al., 2025). The impacts of this extend beyond transcriptomics, specifically providing deeper insights into the relationship between genetic variation and biological function in rare diseases (Grytten et al., 2019; Wang et al., 2024).

In conclusion, graph-based approaches represent a transformative shift towards truly equitable precision medicine that delivers accurate, clinically actionable insights across all populations regardless of their ancestral background or genetic diversity.
